# Subtyping on Live Lymphoma Cell Lines by Raman Spectroscopy

**DOI:** 10.3390/ma15020546

**Published:** 2022-01-12

**Authors:** Klytaimnistra Katsara, Konstantina Psatha, George Kenanakis, Michalis Aivaliotis, Vassilis M. Papadakis

**Affiliations:** 1 Institute of Molecular Biology and Biotechnology, Foundation for Research and Technology-Hellas, N. Plastira 100, GR-70013 Heraklion, Greece; klytaimnistra_katsara@imbb.forth.gr (K.K.); konstantina_psatha@imbb.forth.gr (K.P.); aivaliot@imbb.forth.gr (M.A.); 2 Department of Chemistry/Biochemistry Section, University of Crete, Andrea Kalokerinou, GR-71500 Heraklion, Greece; 3 Institute of Electronic Structure and Laser, Foundation for Research and Technology-Hellas, N. Plastira 100, GR-70013 Heraklion, Greece; gkenanak@iesl.forth.gr

**Keywords:** Raman spectroscopy, PCA, subtyping, cell lines, non-Hodgkin lymphoma, Hodgkin lymphoma, temperature

## Abstract

Raman spectroscopy is a well-defined spectroscopic technique sensitive to the molecular vibrations of materials, since it provides fingerprint-like information regarding the molecular structure of the analyzed samples. It has been extensively used for non-destructive and label-free cell characterization, particularly in the qualitative and quantitative estimation of amino acids, lipids, nucleic acids, and carbohydrates. Lymphoma cell classification is a crucial task for accurate and prompt lymphoma diagnosis, prognosis, and treatment. Currently, it is mostly based on limited information and requires costly and time-consuming approaches. In this work, we are proposing a fast characterization and differentiation methodology of lymphoma cell subtypes based on Raman spectroscopy. The study was performed in the temperature range of 15–37 °C to identify the best cell measurement conditions. The proposed methodology is fast, accurate, and requires minimal sample preparation, resulting in a potentially promising, non-invasive strategy for early and accurate cell lymphoma characterization.

## 1. Introduction

Human lymphoma represents a diverse group of blood cancers emanating from lymph nodes [1]. There are two major categories of lymphoma: Hodgkin lymphoma (HL; B-cell origin) and Non-Hodgkin lymphoma (NHL; B-, T-, NK- cell origin) [2]. Under the microscope, HL are mainly distinguished from NHL by the presence of multinucleated Reed-Sternberg cells (RS cells). However, occasionally, RS cells with similar or identical morphology and phenotype are seen in both HL and NHL [3,4], posing significant diagnostic and therapeutic challenges.

Classical Hodgkin lymphoma (cHL) and mantle cell lymphoma (MCL) are both heterogeneous B-cell derived lymphomas, with distinctive clinical presentation, histological and molecular signatures. cHL, one of the two major categories of Hodgkin lymphoma (HL) accounting for approximately 30% of all lymphomas [5], is considered as a highly curable malignancy, however this is not the case for elderly patients [6]. Mantle cell lymphoma (MCL), a rare and typically aggressive form of NHL representing about 3–10% of adult-onset NHL in Western countries, is often diagnosed at a late stage during biopsy [7]. Therefore, improvements in early diagnosis will most likely advance cHL and MCL management and survival rate [8].

Raman spectroscopy is a promising, non-destructive label-free technique for the lymphoma cell early detection in the blood and its biochemical profile. It is a vibrational spectroscopy method, with proven applications in a variety of scientific fields for diagnostic purposes. Raman spectroscopy has been applied in cancer diagnosis [9,10] in tissues, [11,12] and in cells [8]. Cell subtyping can further be supported by PCA which is a standard technique used in Raman spectroscopy [13]. Raman spectroscopy analysis and imaging of nucleus area in live HL and NHL cells could potentially provide more accurate and clear results in regard to the different lymphoma “fingerprinting”, distinguishing different lymphoma subtypes [14].

Previously, scientists distinguished non-Hodgkin lymphoma B-cells from normal B-cells using Raman spectroscopy to demonstrate the unique fingerprint of each cell line [8]. B-NHL Burkitt’s lymphoma cell lines (Ramos and CA46) were cultured and normal B-cells were isolated from peripheral blood. A Micro-Raman RXN system was used for the Raman measurements, whereby cells were placed directly on aluminum reflective slides cleaned with methanol before and after the measurements. Each measurement collected the Raman signal for 60 s with 10 mW laser power in the range from 600 to 1800 cm^−1^, providing the biological information concerning the cells’ constituents. Raman spectroscopy analysis provided fingerprints for DNA/protein concentrations and saccharide bonds, which were difficult to distinguish. For this reason, principal component analysis (PCA) was performed to detect the differences between the cell types. In conclusion, the Raman spectroscopy fingerprints of normal B-cells and B-NHL cells had similar peaks and phenotypes, which could be assigned to cellular constituents (DNA/RNA, amino acids, lipids, carbohydrates).

Based on our knowledge and the available literature, a very limited number of studies have used Raman spectroscopy in lymphoma and there is no priori work on the analysis and comparison of HL and NHL using Raman spectral characteristics. This study focuses on comparing two model lymphoma cell lines (MDA-V/HL and JMP-1/MCL; NHL) to evaluate Raman spectroscopy as a tool for the differentiation of the two lymphoma subtypes, screening them for characteristic Raman shifts. Raman spectra were acquired from the intracellular nucleus area in live cells. Tests were performed under different temperatures, ranging from 15 °C to 37 °C, investigating the best cell measurement conditions and confirming that the proposed methodology can be applied under normal cell growth conditions (37 °C). Subsequently, principal component analysis (PCA) was used to detect the differences between the complex Raman signals. Results indicate that HL and NHL cell lines can be differentiated at room temperature.

## 2. Materials and Methods

### 2.1. Cell Lines, Culture Conditions and Sample Preparation

Two B-cell lymphoma cell lines harboring wild type *p53* (wtp53), MDA-V/HL (cHL), and Epstein Barr virus ((EBV)-positive cHL) and JMP-1/MCL NHL (MCL) were used. Both cell lines were maintained under standard conditions of exponential growth, as described previously (RPMI 1640 medium supplemented with 15% fetal bovine serum, (FBS), incubated at 37 °C in a 5% CO_2_ humidified atmosphere) [15]. Cell viability (>90%) was assessed using Trypan blue staining prior to Raman measurements. Cells were washed with phosphate buffered saline (PBS), re-suspended in phenol-free RPMI 1640, and 100,000 cells were used for further analysis, where 20 μL of the resulting cell suspension was placed directly on a clean CaF_2_ microscope slide.

**Figure 1:**
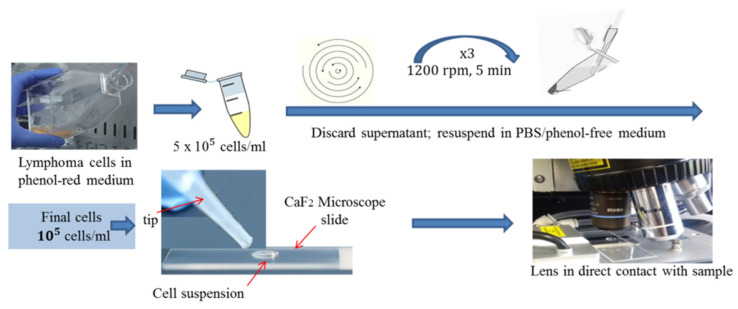
Sample preparation process.

A schematic of the procedure followed during live cell Raman spectroscopy analysis is depicted in Figure 1.

The measured cells, had a diameter approximately of: 10–25 μm for the MDA-V/HL, and 12–14 μm for the JMP-1/MCL cell lines.

All sample preparation was performed under a ducted fume hood with ventilation to outside, which provides the most user protection mostly for chemically dangerous fumes, and close to the Raman microscope a Biological Safety Cabinet (BYKG-I, Biobase, Shandong, China), enhanced with UV lamp for sterilization, suitable for work involving low to moderate risk agents (Biosafety Levels 1, 2 and 3). Unlike a conventional fume hood, the HEPA filter in the Class I Biosafety Cabinet protects the environment by filtering air before it is exhausted, while with the negative pressure, personnel protection is made possible by constant movement of air into the work area. As a result, the “BIOBASE BYKG-I” Class I Biological Safety Cabinet provides protection for the user and surrounding environment, but no protection for the sample being manipulated.

### 2.2. Instrument and Measurement Preparation

Raman measurements were performed using a modified LabRAM HR Raman Spectrometer from HORIBA Scientific.

#### 2.2.1. Instrument Description 

LabRAM HR Raman Spectrometer (HORIBA Scientific, Lille, France) is a confocal Raman microscope. The Raman excitation laser line had a central wavelength at 532 nm and a laser output power of ~100 mW. The objective lens used was an Olympus 60× water immersion lens with a numerical aperture (NA) of 1.2 and a working distance of 280 μm (UPLSAPO60XW/1.2, Olympus), which was in direct contact with the sample. The resulting maximum laser power (100%) on the sample under the aforementioned setup was measured to be 32 mW with a laser spot of 0.7 μm (0.54 μm in theory), with an axial length about 0.6 μm (0.47 μm in theory). A grating of 600 groves/mm was used that resulted to a Raman spectral resolution of around 1 cm^−1^. The Raman signal detector was the Syncerity CCD Deep Cooled Camera by Horiba, operating at −50 °C. A temperature-controlled stage PE120-XY (Linkam, Tadworth, United Kingdom) was coupled with the microscope stage to ensure the sample’s temperature control and stability. This stage incorporates a PT100 temperature sensor (Linkam, Tadworth, United Kingdom), that provides feedback to the controller, achieving control and stability in temperature of +/−0.1 °C. 

Instrument calibration was performed before each experiment. Spectral calibration was performed with a Si reference sample, presenting a single peak at 520.7 cm^−1^. Validation of the spectral calibration was further achieved with the substrate used for the samples, which was a Raman grade CaF_2_ microscopic slide obtained by Crystran, which has a single Raman peak at 321 cm^−1^. The substrate Raman spectrum was tested, and no other Raman peaks appeared following the same experimental conditions.

#### 2.2.2. Acquisition Settings 

The microscope was setup to acquire Raman signals in the spectral range between 300 and 3150 cm^−1^, which corresponds to two spectral windows with the current grating. Laser operated at maximum intensity (100%). Measurements were acquired in the center of each cell (nucleus). Acquisition time was set to 5 s with a spectral accumulation of 3 spectra/point. This resulted to a total acquisition time of around 40 s per point. These acquisition settings were tested before the experiment to ensure that cells were not visibly damaged by the laser power during the exposure of 40 s, through the bright-field microscope.

#### 2.2.3. Measurement Process

20 μL of cell’s suspension was transferred from the Eppendorf tube to the CaF_2_ substrate. The water immersion lens was in direct contact with the cell suspension, achieving maximum efficiency of the Raman spectrum measurement. A relaxation time of approximately 5 min was allowed, for temperature stabilization and for the cells to sink and relax on the substrate surface. When the objective lens focused in the middle of each cell, it created a thin layer of cell suspension of approximately 300 μm thickness between the substrate’s surface and the objective lens. 

From each cell, one measuring point was observed, being the center of the cell, aiming at the cell nucleus. It is worth noting that, following each measurement, a second measurement was acquired, being the surrounding area of the cells, which was used for background subtraction.

Following each measurement, we used a cleaning solution of 85% n-hexan and 15% isopropyl alcohol to clean the objective lens and ethanol for all the microscopic slides before proceeding to the next measurement. The users wore gloves and FFP2/N95 masks when handling the samples. The remaining samples were sent back to the laboratory for proper treatment to be discarded.

#### 2.2.4. Experiments Performed

Raman measurements of lymphoma cells were performed always monitoring temperature, keeping it constant during acquisition. Cell measurements were performed at six different temperatures (37, 30, 25, 20, 18, 15 °C). The temperature range was selected based on the higher and lower limits set for this experiment. The upper limit corresponded to the normal physiological temperature cells thrive at (T = 37 °C). The lower limit (T = 18 °C) was the temperature where the threshold existed between liquidation of the environmental humidity and evaporation. The temperature range between these two limits was covered in steps of 5 °C (T = 30 °C, 25 °C, and 20° C). Since T = 35 °C was very close to the upper limit, it was excluded from the temperature list. T = 15 °C was used as an extra temperature value to test Raman signals and cell viability.

Four independent experiments were performed, with two biological repeats for every cell line (BRx), and with x defining the number of the experiment. BR1 and BR2 correspond to JMP-1/MCL NHL cell measurements. In BR1, cells were measured under 37, 30, 18, and 15 °C temperatures. In BR2, cells were measured under 30, 25, 20, and 15 °C temperatures. BR3 and BR4 refer to MDA-V/HL cell measurements. In BR3, cells were measured under 37, 30, 25, 20, 18, and 15 °C temperatures. In BR4, cells were only measured under 15 °C. Data from all four experiments under all temperatures were included in the final analysis.

#### 2.2.5. Raman Spectral Database

Through the analysis of the Raman signals and the peaks identified, a database was constructed from information derived from literature in order to translate Raman spectral findings to biological information [16,17,18]. The constructed database with the Raman peaks and related assignments can be found in Table S1. Raman peak assignments selected included both nucleus and nuclear membrane information, since our detection volume could have interfering signals from both. Nucleus houses nucleic acids (DNA/RNA), proteins, phospholipids, various phosphate compounds, and several inorganic compounds. The nucleus is surrounded by a nuclear membrane, which consists of phospholipids and proteins (forming nuclear pores). In the left column of the table, we present the Raman peak wavenumber. When a temperature dependence was observed in the Raman peaks, an asterisk was placed next to the corresponding wavenumber for discrimination. In the second column, the Raman peak assignments are presented. Additionally to the asterisk, a color coding (red color) is used to indicate the temperature-based Raman peaks and assignments.

### 2.3. Raman Spectra Processing and Analysis

Raw Raman spectral data acquired from the Raman microscope underwent the following processing methodology: (a) cosmic rays were removed by an internal function *Despike* (LabSpec LS6, Horriba); (b) background signal was acquired from the cell suspension in the neighborhood area outside of the cell; (c) background signal was subtracted from the raw Raman spectral data. A typical example of this process can be found in the supporting (Figure S1). No further processing (smoothing, or noise cancellation processing) was performed in the Raman spectral data to ensure minimum effect in the analysis.

Due to the size of our experimental data, principal component analysis was chosen as a potential unsupervised discrimination methodology of the cellular populations. This is the standard technique used for discrimination approaches [19]. We could not perform supervised approaches like (PLS-DA), particularly given that the requirement for the training data would lead to a highly undertrained model [20]. In the case of a significantly larger data set, in the range of hundreds, PLS-DA would be one good methodology for this analysis, and to potentially examine the effectiveness of other multivariate classification techniques that could lead to cell line classification. 

PCA was performed using a tool (Raman PCA Tool, by Patrizio Candeloro [21]) publically available through (access date: 17 November 2021, https://sourceforge.net/projects/ramantoolset/), for the comparison of HL and NHL cells, but also for comparison between the different experiments (BR). When we carry out a PCA analysis, our aim is to find the principal component spectral characteristics which indicate the greatest differentiation and/or clustering among the selected samples.

## 3. Results and Discussion

A full Raman Spectrum (300–3150 cm^−1^) was acquired from a total number of 60 live cells from two distinct lymphoma cell lines (JMP-1/MCL; NHL and MDA-V/HL). In this experiment, we measured various points (cell nucleus, cell cytoplasm, cell membrane, and extracellular space). Raman signals from the nucleus area gave us distinct peaks that were strongly repeatable, and we can be certain that they correspond to cell variations. As discussed in the experimental section, our experimental procedure had a spot size of approximately 0.8 um, which is significantly larger than the cell membrane dimensions (~50 nm). This caused significant signal interference deriving from the cytoplasm and/or the extracellular space. Further, measurements in the membrane as well as in the cytoplasm were affected by the cell’s mobility, causing more interference with either membrane or nucleus. This resulted in measurements with low repeatability, so we decided to exclude them from this work. Concluding, Raman signals acquired from the nucleus area presented high repeatability that we could trust, so we used only those to compare and analyze the differences between JMP-1/MCL, NHL, and MDA-V/HL cells. Raman spectral data were processed as described in Section 2. Hence, we show that there is a distinct differentiation between the Raman signals extracted from the nuclei of the two cell lines. 

### 3.1. JMP-1/MCL NHL and MDA-V/HL Cell Lines Comparison

The comparison of JMP-1/MCL NHL and MDA-V/HL cell lines was performed for all experiments (BR1, BR2, BR3, BR4)

**Figure 2:**
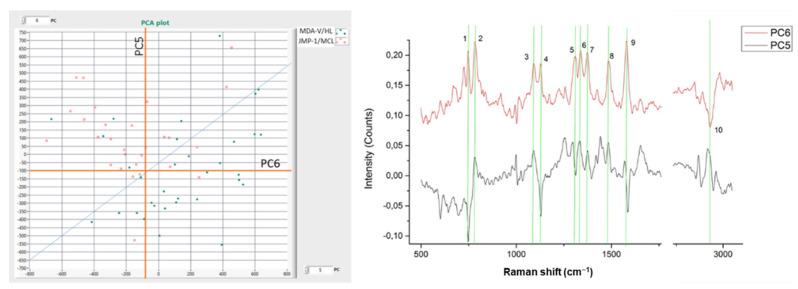
(**left**) Comparison (*n* = 60) between MDA-V/HL and JMP-1/MCL NHL cell lines; (**right**) PC Raman spectra showing the cell lines spectral differences.

In Figure 2, the PCA plot, the average Raman spectra, and the associated Raman spectral differences are depicted, deriving from the total number of cells measured in our experiments.

In particular, from the PCA plot, we see that PC5 separates the cell lines with a cell differentiation efficiency (CDE) of 71.66%, and PC6 with a CDE of 68.33%, resulting in a higher CDE of 81.66% when both PCs (5 and 6) are taken into account.

From the resulting PCs (PC5 and PC6), we calculated their spectral curves and identified the major peaks and common lines that are responsible for the differentiation of the two cell lines. These findings are depicted with a green line in Figure 2, and their value is presented in Table 1. This table shows the common line wave numbers and their assignments from the Raman spectral database.

In Figure 2, it is shown that the cell differentiation has a CDE of 81.66%. 

As described in the experimental process we mainly performed measurements at 15 °C. Raman peak differences affected by the temperature changes were identified and described in the related section. Further, to better understand if the CDE was affected by other parameters except the cell lines subtype differences, we tested three different hypotheses based on the individual experimental BR we performed. Initially, we tested if the two (BR1 vs. BR2) that involve the same cell line type differ, and second if the two (BR1 vs. BR3, 4) and (BR2 vs. BR3, 4) present different CDE. Table 1 presents the most important signaling differences between HL and NHL lymphoma cell lines. The Raman peaks that are affected by temperature are highlighted (in red color and with a superscript). As it is shown, six out of 10 of the major Raman peaks are not affected by temperature. This fact is important since it can support the hypothesis that cell subtyping can be performed independently of the temperature allowing measurements in room temperatures.

Additionally, we repeated the PCA by performing three main processing tasks for better differentiation between the two cell lines. The first processing was to correct the baseline with a polynomial function. The second processing was mean centering and the third was to perform variance adjustment. Results showed no significant improvement on the differentiation between the two cell lines. The results from this PCA are presented in Figures S2 and S3. As expected, due to the high variance between each data set, the mean centering did not affect the results as much. The fact that PC5 and PC6 indicate potential discrimination of the cell lines, in both analyses, indicates that the coherent differences are very small, though potentially significant, as such enabling unsupervised analysis. Thus, we considered using the data that were background subtracted and not baseline corrected as it may help shoulder peaks to have better significance.

### 3.2. Comparison of Experiments BR1 vs. BR2 of JMP-1/MCL NHL Cell Line

**Figure 3:**
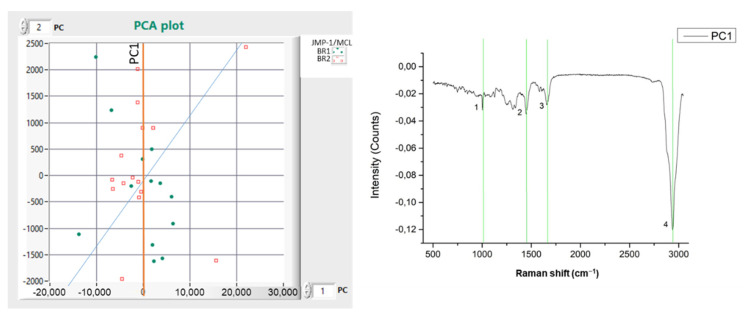
(**left**) PCA plot between measurements of two distinct experimental periods of from the same cell line. Green is BR1, Red is BR2 (JMP-1/MCL NHL); (**right**) PC Raman spectra showing the cell line differences.

This analysis showed that the two cell lines had a low CDE. In particular, the best CDE was achieved under PC1 (Figure 3), where differentiation occurred in four Raman peaks (Table 2) and mainly in the peak of 2938 cm^−1^. In all other PCs, the CDE was lower than 50%.

As a result, we conclude that there is no clear separation found between BR1 and BR2 at the Raman peaks identified as important for the cell lines differentiation described in Table 1. 

The average Raman spectra from BR1 and BR2 experiments are presented in Figure S4. Further, the major Raman peaks identified from the PCA are presented in the following Table 2.

Comparing the Raman spectral differences of BR1 and BR2 identified in Table 2 with the Raman peaks identified as important for the differentiation of the cell lines described in Table 1, we see that the four Raman peaks in Table 2 are not significant for the cell differentiation.

### 3.3. Subtyping BR1 vs. BR3, BR4 (JMP-1/MCL NHL vs. MDA-V/HL)

**Figure 4:**
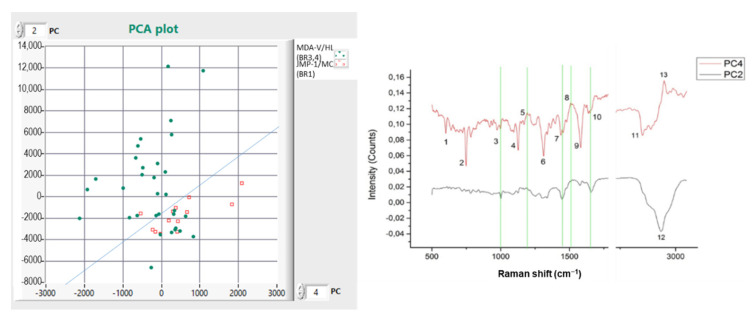
(**left**) Comparison between experiments BR1 (JMP-1/MCL NHL) and BR3,4 (MDA-V/HL) cell lines; (**right**) PCA Raman spectra showing the cell lines differences.

As presented in Figure 4, we see that the two cell lines are separated with a CDE of 77.27%. 

Further, the major Raman peaks identified are presented in Table 3.

### 3.4. Subtyping BR2 vs. BR3,4 (JMP-1/MCL NHL vs. MDA-V/HL)

**Figure 5:**
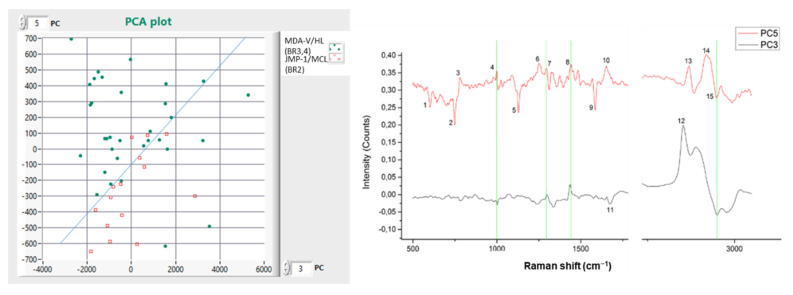
(**left**) Comparison between experiments BR2 (JMP-1/MCL NHL) and BR3,4 (MDA-V/HL) cell lines; (**right**) PCA Raman spectra showing the cell lines differences.

As presented in Figure 5, we see that the two cell lines are separated with a CDE of 81.25%. 

Further, the Raman peaks were found and are presented in Table 4.

### 3.5. Cell Line Differences Due to Temperature

The normal temperature of the human cells is constant at Τ = 37 °C, so live cell measurements should be performed at this temperature. Unfortunately, we faced two major problems in performing Raman measurements under this temperature. First, due to the long measurement period (~1 h), the cell’s suspension medium (RPMI phenol free medium) began evaporating, and it was impossible to complete a full experiment. Second, live cells were moving from their initial position during each measurement, indicating a movement of more than 50% of their size.

In order to succeed in acquiring nuclei representative results, we decided to work under a lower sample temperature (15 °C), where both problems were minimized. In particular, the evaporation of the cell’s suspension medium was the minimum, with no visible evaporation of the RPMI after 1 h. The temperature of 15 °C was selected following tests for evaporation and liquidation of the environmental humidity. By maintaining a low temperature, cells were found to be relaxed, probably since they received lower energy amounts from their environment, losing their mobility, which allowed more accurate Raman measurements. 

To ensure that this solution of the new temperature of 15 °C had no potential negative effect in our cell subtype differentiation, in the previously identified Raman spectral characteristics, we performed experiments in six different temperatures ranging from 15 to 37 °C for both cell lines. The identified Raman signal differences were compared with the Raman spectral database to verify that they do not contribute to the cell lines differentiation. Further, bright-field images were acquired before and after each measurement to detect any movements of the cell, or any defocusing due to thermal changes caused by laser absorption from the sample.

As mentioned, the effect of temperature on Raman measurements was tested in both cell lines. The most significant Raman peaks identified that are affected by temperature together with their assignments are presented in the following related tables.

It must be mentioned that in the beginning of the experiments we tried to measure at T = 15 °C, which proved to be impossible because of the high moisture content of the environment (54%) that resulted in liquidation and expansion of the cell suspension. To solve this, we raised the temperature at T = 18 °C. In other experimental days where humidity was lower (40%), we were able to measure at T = 15 °C. For both temperatures (15 and 18 °C), a measurement duration of 1.5 h presented no visual losses on the sample’s suspension.

#### 3.5.1. MDA-V/HL Cell Line Temperature Differences (T_n_-T_37_ MDA-V/HL)

**Figure 6:**
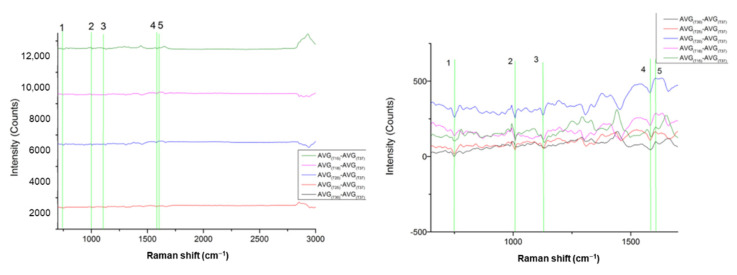
(**left**) Raman signal differences due to temperature (Τn-T37 plot); (**right**) Magnification of Raman signal differences.

In Figure 6, the average Raman signal differences T_n_-T_37_ from all BR3 experiments are presented. In green, the most significant differences with a linear variation versus temperature are identified and depicted.

In Table 5, differences between the identified Raman peaks are presented.

#### 3.5.2. JMP-1/MCL NHL Cell Line Temperature Differences (T_n_-T_37_ JMP-1/MCL NHL)

**Figure 7:**
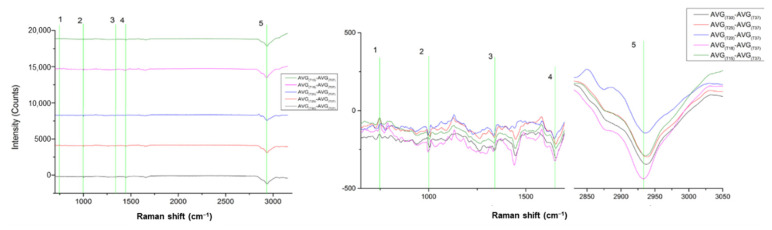
(**left**) Raman signal differences due to temperature (Τn-T37 plot); (**right**) Magnification of Raman signal differences.

In Figure 7, the average Raman signal differences T_n_-T_37_ from all BR1, 2 experiments are presented. In green, the most significant differences with a linear variation versus temperature are identified and depicted.

In Table 6, differences in the identified Raman peaks are presented.

In all tables, we highlight in red the Raman peaks that were also similar to the Raman peaks found to be contributing to the cell differentiation derived from the PCA analysis. This tells us that the red-highlighted peaks cannot be taken in account for the discrimination of the two cell lines. In contrast, the rest of the Raman peaks are not found in the temperature-based experiments, so they will be considered as significant for the cell type discrimination.

The analysis of the temperature differences between the same cell line showed that there are changes occurring for the Raman peak shifts. In particular, the average Raman spectra from J-MP1 and MDA-V cell lines from all measurements in all temperatures with their standard deviation are presented in Figure S5. Further, in the Tables S2 and S3 (for MDA-V/HL and JMP-1/MCL NHL, respectively), we show the intensity variations versus the temperature changes from the normal temperature of T = 37 °C. Moreover, Raman peak shifts range is presented in the second line of the tables.

### 3.6. Cell Viability

Based on the overall experiments we performed, we had an average viability of approximately 90%. Cell viability originally was confirmed by cell counting with trypan blue and bright-field images captured the shape and morphology for the two cell lines of our study over experimental time and temperature, monitoring the status of the cells.

During one hour of Raman measurements, the viability of lymphoma cells depended on the temperature that was regulated by Linkam. A significant number of cells at higher temperatures (T = 37 °C, T = 30 °C and T = 25 °C) were lysed after Raman measurement, in contrast to cells at lower temperatures (T = 20° C, T = 18 °C T = 15 °C), which remained alive. We believe that this is due to the fact that, at higher sample temperatures, cells receive higher energy amounts from their environment. This combined with the high energy received from the excitation laser source is above the acceptance threshold of the cells. However, all examined cells considered in this work were alive before and after Raman measurements. Randomly selected cell images after laser irradiation are presented in Figure S6. Generally, we realized that, after 1 h of measurement at T = 37 °C, only 10% of cells were still alive in contrast to T = 15 °C, where 99% of cells remained alive. Furthermore, cells did not present any visible damage during the measurements with the high laser illumination (32 mW for 40 s).

## 4. Conclusions

In this work, we show that cell line subtyping can be achieved by means of Raman spectroscopy. Furthermore, in Table 2, we present the Raman peaks that were identified as significant for this purpose. 

An efficient and simple Raman spectral measurement methodology is presented that enabled us to achieve fast and robust measurements.

It was shown that temperature affects the Raman spectrum of the cell line nuclei in specific Raman peaks but it did not contribute to cell line differentiation (subtyping).

Results indicate that Raman spectroscopy is a reliable tool for cell line differentiation.

Future work should include a larger sample size for each cell line, as well as multiple other cell lines. The implementation of machine learning algorithms would enable better cell line characterization and possibly identification. This has the potential to lead to faster, better, and earlier lymphoma diagnosis.

## Figures and Tables

**Table 1:** Most important Raman signaling differences between HL/cHL and MCL/NHL lymphoma cell lines, with the central wavelength of the Raman peaks and the related assignments. Red color and asterisk superscript correspond to the temperature-dependent Raman peaks.

No	PC6 Peaks (cm^−1^)	PC5 Peaks (cm^−1^)	Assignment
1 *	747	748	747 cm^−1^ → CH_2_ rocking, L-Phenylalanine [22], 748 cm^−1^ → DNA, ring breathing of pyrimidine of T [23]
2	783	782	Phosphodiester, Cytosine, Thymine, Uracil
3	1091	1091	1090 cm^−1^ → Symmetric phosphate stretching vibrations, 1092–1093 cm^−1^ → Phosphodioxy
4 *	1127	1129	v(C-N) stretching (lipids, 1127 cm^−1^ → proteins), ν(C-C) skeletal of acyl backbone in lipid (1129 cm^−1^ → trans conformation)
5	1310	1312	1309 cm^−1^ → CH_3_/CH_2_ twisting or bending mode of lipid/collagen, 1313 cm^−1^ → CH_3_CH_2_ twisting mode of collagen/lipid
6 *	1337	1334	1334 cm^−1^ → DNA/RNA purine bases, Guanine, Adenine), proteins (1337 cm^−1^ → amide III, CH_2_ wagging vibrations from Glycine backbone and proline side chain, L-Histidine, L-Tryptophane, L-Glutamate)
7	1373	1375	T, A, G (ring breathing modes of the DNA/RNA bases), Acetyl coenzyme A [22]
8	1484	1484	1480–1575 cm^−1^ → Amide II (largely due to a coupling of C-N stretching and in-plane bending of the N-H group 1485 cm^−1^ → G, A (ring breathing modes in the DNA bases) Nucleotide acid purine bases (guanine and adenine), Purine rings (guanine) [24] 1483 cm^−1^ → CG (C4 me) associated with CH_2_ and CH_3_ bending and stretching vibrations, CG (C4 me) [24]
9 *	1578	1586	1586 cm^−1^ → DNA/RNA, 1578 cm^−1^ → Guanine (N3), Guanine, adenine
10	2928	2919	CH_2_ asym stretches and CH stretches in lipids and proteins

**Table 2:** Major Raman peaks found in PC1 between BR1 and BR2 analysis.

No	PC2 Peaks (cm^−1^)	Assignment
1	1002	Proteins, C-C aromatic ring stretching (collagen assignment)
2	1447	CH_2_ bending mode of proteins and lipids, CH_2_ deformation (protein vibration), a marker for protein concentration, δas (CH_3_) δ(CH_2_) of proteins
3	1655	Proteins, Lipids, ν(C=O) amide I, α-helix, C=C lipid stretch
4	2938	CH_2_ asym stretches and CH stretches in lipids and proteins

**Table 3:** Major Raman peaks found in PCA between BR1 and BR3, 4. In red color and with an asterisk superscript the temperature dependent Raman peaks are presented.

No	PC4 Peaks (cm^−1^)	PC2 Peaks (cm^−1^)	Assignment
1	601	-	Nucleotide conformation
2 *	749	-	749 cm^−1^ → Symmetric breathing of tryptophan
3	999	1001	999 cm^−1^ → ν_45_(C-C), observed in the spectra of single human Red blood cell (RBC), 1001 cm^−1^ → Symmetric ring breathing mode of phenylalanine
4 *	1127	-	v(C-N) stretching (proteins)
5	1194	1191	1191 cm^−1^ → L-Valine, acetoacetate, 1194 cm^−1^ → L-Proline [22], 1185–1300 cm^−1^ → Antisymmetric phosphate vibrations
6	1312	-	1313 cm^−1^ → CH_3_CH_2_ twisting mode of collagen/lipid
7	1446	1445	δ(CH_2_), δ(CH_3_) protein (collagen) and lipid (phospholipids) assignment (CH_2_ bending mode being of diagnostic significance), CH_2_ deformation (1446 cm^−1^)
8	1509	1502	1499 cm^−1^ → C-C stretching in benzenoid ring, 1510 cm^−1^ → Cytosine, A (ring breathing modes in the DNA bases
9 *	1582	-	C=C stretching in Phenylalanine, hydroxyproline
10	1650	1656	1650 cm^−1^ → amide I (C=C) absorption, 1656 cm^−1^ → cis phospholipids, Carbonyl stretch (C=O)
11	2851	-	CH_3_ symmetric stretch of lipids
12	-	2934	2934 cm^−1^ → CH_2_ asym stretches and CH stretches in lipids and proteins
13	2949	-	CH_3_ stretching vibrations

**Table 4:** Major Raman peaks found in PCA between BR2 and BR3, 4. In red color and with an asterisk superscript the temperature dependent Raman peaks are presented.

No	PC5 Peaks (cm^−1^)	PC3 Peaks (cm^−1^)	Assignment
1	602	-	Nucleotide conformation
2 *	749	-	Symmetric breathing of tryptophan
3	779	-	DNA/RNA
4	999	1000	900 cm^−1^ → ν_45_(C-C), observed in the spectra of single human Red blood cell (RBC), 1000 cm^−1^ → Phenylalanine, Bound and free NADH
5 *	1129	-	ν(C-C) skeletal of acyl backbone in lipid → trans conformation
6	1251	-	Guanine, cytosine (NH_2_)
7	1298	1298	1220–1300 cm^−1^ → Amide III (arising from coupling of C-N stretching & N-H bonding-can be mixed with vibrations of side chains), amide III (L-Arginine [22]), CH bend in Lipids (Palmitic acid), Acyl chains
8	1441	1435	1435 cm^−1^ → Thymine (weak) [22], 1420–1481 cm^−1^ → DNA/RNA, Guanine, Adenine, 1441 cm^−1^ → CH_2_ scissoring and CH_3_ bending in lipids, Cholesterol and its esters, C-H bending mode of accumulated lipids in the vecrotic core of the atheromatous plaque
9 *	1586	-	DNA/RNA
10	1653	-	1653 cm^−1^ → Lipid (C=C stretch, Ascorbic acid [22])
11	-	1672	C=C stretch, Amide I band (C=O stretch coupled to a N-H bending)
12	-	2852	CH_3_ symmetric stretch of lipids
13	2869	-	CH_2_ asym stretches and CH stretches in lipids and proteins, CH_2_ symmetric stretch of lipids
14	2919	-	CH_2_ asym stretches and CH stretches in lipids and proteins, CH_2_ asymmetric stretch of lipids and proteins, CH_3_ stretching vibration
15	2947	2950	CH_3_ stretching vibrations

**Table 5:** Major Raman peaks found in BR3 under different temperatures. In red color and with an asterisk superscript the temperature dependent Raman peaks are presented.

No	Peaks (cm^−1^)	Assignment
1 *	749	Symmetric breathing of tryptophan
2	1008	Proteins (L-Serine) [22], Phenylalanine, ν(C-O), ν(C-C)
3 *	1125–1134	Adenine (1125, 1134 cm^−1^ [22]) and v(C-N) stretching (lipids, 1127 cm^−1^ → proteins), ν(C-C) skeletal of acyl backbone in lipid (1129 cm^−1^ → trans conformation), Phospholipid structural changes (trans versus gauche isomerism), Acyl chains, Palmitic acid
4 *	1581–1586	DNA/RNA (1586 cm^−1^), Phenylalanine, hydroxyproline (1582 cm^−1^ → C=C stretching)
5	1604	1600–1800 cm^−1^ → Amide I band of proteins; due to C=O stretching Amide I (which is due mostly to the C=O stretching vibrations of the peptide backbone; has been used the most for structural studies due to its high sensitivity to small changes in molecular geometry and hydrogen bonding of peptide group) 1602 cm^−1^ → Phenylalanine, δ(C=C), phenylalanine (protein assignment) 1603 cm^−1^ → C=C in-plane bending mode of phenylalanine and tyrosine, Ring C-C stretch of phenyl,1605 cm^−1^ → Cytosine (NH_2_)

**Table 6:** Major Raman peaks found in BR1, BR2 under different temperatures. In red color and with an asterisk superscript the temperature dependent Raman peaks are presented.

No	Peaks (cm^−1^)	Assignment
1 *	745–748	700–745 cm^−1^→ ν(C-S) trans (amino acid methionine), 747 cm^−1^→ CH_2_ rocking, L-Phenylalanine [22], 748 cm^−1^ → DNA, ring breathing of pyrimidine of T [23]
2	999–1001	999 cm^−1^ → ν_45_(C-C), observed in the spectra of single human Red blood cell (RBC), 1001 cm^−1^ → Symmetric ring breathing mode of phenylalanine
3 *	1333–1341	Polynucleotide chain (1334 cm^−1^ → DNA/RNA purine bases, Guanine, Adenine), proteins (1337 cm^−1^ → amide III, CH_2_ wagging vibrations from Glycine backbone and proline side chain, L-Histidine, L-Tryptophane, L-Glutamate), CH_3_CH_2_ wagging mode of collagen, CH protein deformation
4	1441–1446	1441 cm^−1^ → CH_2_ scissoring and CH_3_ bending in lipids, Cholesterol and its esters, C-H bending mode of accumulated lipids in the vecrotic core of the atheromatous plaque, 1446 cm^−1^ → δ(CH_2_), δ(CH_3_) protein (collagen) and lipid (phospholipids) assignment (CH_2_ bending mode being of diagnostic significance), CH_2_ deformation
5	2934	CH_2_ asym stretches and CH stretches in lipids and proteins

## Data Availability

All data are available upon request. The data presented in this study are available on request from the corresponding author.

## Supplementary Materials

The following supporting information can be downloaded at: https://www.mdpi.com/article/10.3390/ma15020546/s1, Table S1: Total list of Raman peak assignments. In red color and with an asterisk in superscript the temperature dependent Raman peaks are presented; Figure S1: Demonstration of the Raman spectral background subtraction. Spectra are presented with an offset between them, to allow better visualization of the spectral background changes; Figure S2: PCA of PC1 and PC2 JMP-1/MCL NHL and MDA-V/HL cell lines comparison; Figure S3: PCA of PC1 and PC2 JMP-1/MCL NHL and MDA-V/HL cell lines comparison; Figure S4: Average Raman spectra from BR1 (left) and BR2 (right) (JMP-1/NHL) experiments; Figure S5: Average Raman spectra from J-MP1 and MDA-V cell lines from all measurements in all Temperatures. In shadow the related SD of each average is presented; Table S2: Major Raman bands identified as significant under different temperatures for the MDAV/HL cell line. In red color and with a star superscript the temperature dependent Raman peaks are presented; Table S3: Major Raman bands identified as significant under different temperatures for the JMP1/MCL cell line. In red color and with a star superscript the temperature dependent Raman peaks are presented; Figure S6: Randomly selected cell images from bright-field microscope. In the upper and bottom line JMP-1/NHL and MDA-V/HL cell line images are presented respectively, monitoring indistinguishable morphological differences following a successful completion of the measurement.

## References

[1] Steliarova-Foucher E., Stiller C., Lacour B., Kaatsch P. (2005). International Classification of Childhood Cancer, third edition. Cancer.

[2] Harris N.L., Jaffe E.S., Diebold J., Flandrin G., Muller-Hermelink H.K., Vardiman J. (2000). Lymphoma classification—From controversy to consensus: The R.E.A.L. and WHO Classification of lymphoid neoplasms. Ann. Oncol..

[3] Caleo A., Sanchez-Aguilera A., Rodriguez S., Dotor A.M., Beltran L., de Larrinoa A.F., Menarguez F.J., Piris M.A., Garcia J.F. (2003). Composite Hodgkin lymphoma and mantle cell lymphoma: Two clonally unrelated tumors. Am. J. Surg. Pathol..

[4] Kramer S., Uppal G., Wang Z.X., Gong J.Z. (2019). Mantle Cell Lymphoma With Hodgkin and Reed-Sternberg Cells: Review With Illustrative Case. Appl. Immunohistochem. Mol. Morphol..

[5] Kuppers R. (2009). The biology of Hodgkin’s lymphoma. Nat. Rev. Cancer.

[6] Driessen J., Visser O., Zijlstra J.M., Lugtenburg P.J., Plattel W.J., Kersten M.J., Dinmohamed A.G. (2021). Primary therapy and relative survival in classical Hodgkin lymphoma: A nationwide population-based study in the Netherlands, 1989–2017. Leukemia.

[7] Jain P., Wang M. (2019). Mantle cell lymphoma: 2019 update on the diagnosis, pathogenesis, prognostication, and management. Am. J. Hematol..

[8] Shiramizu B., Oda R., Kamada N., Garcia M.A., Shieh T., Maeda T.A., Choi S.Y., Lim E., Misra A. (2018). Unique Raman Spectroscopic Fingerprints of B-Cell Non-Hodgkin Lymphoma: Implications for Diagnosis, Prognosis and New Therapies. J. Biol. Med. Sci..

[9] Keshavarz M., Tan B., Venkatakrishnan K. (2018). Label-Free SERS Quantum Semiconductor Probe for Molecular-Level and in Vitro Cellular Detection: A Noble-Metal-Free Methodology. ACS Appl. Mater. Interfaces.

[10] Keshavarz M., Chowdhury A.K.M.R.H., Kassanos P., Tan B., Venkatakrishnan K. (2020). Self-assembled N-doped Q-dot carbon nanostructures as a SERS-active biosensor with selective therapeutic functionality. Sens. Actuators B Chem..

[11] Rau J.V., Marini F., Fosca M., Cippitelli C., Rocchia M., Di Napoli A. (2019). Raman spectroscopy discriminates malignant follicular lymphoma from benign follicular hyperplasia and from tumour metastasis. Talanta.

[12] Lloyd G.R., Orr L.E., Christie-Brown J., McCarthy K., Rose S., Thomas M., Stone N. (2013). Discrimination between benign, primary and secondary malignancies in lymph nodes from the head and neck utilising Raman spectroscopy and multivariate analysis. Analyst.

[13] Lin D., Lin J.Q., Wu Y.A., Feng S.Y., Li Y.Z., Yu Y., Xi G.Q., Zeng H.S., Chen R. (2011). Investigation on the interactions of lymphoma cells with paclitaxel by Raman spectroscopy. Spectrosc. Int. J..

[14] Das R.S., Agrawal Y.K. (2011). Raman spectroscopy: Recent advancements, techniques and applications. Vib. Spectrosc..

[15] Leventaki V., Drakos E., Karanikou M., Psatha K., Lin P., Schlette E., Eliopoulos A., Vassilakopoulos T.P., Papadaki H., Patsouris E. (2014). c-JUN N-terminal kinase (JNK) is activated and contributes to tumor cell proliferation in classical Hodgkin lymphoma. Hum. Pathol..

[16] Talari A.C.S., Movasaghi Z., Rehman S., Rehman I.U. (2015). Raman Spectroscopy of Biological Tissues. Appl. Spectrosc. Rev..

[17] Czamara K., Majzner K., Pacia M.Z., Kochan K., Kaczor A., Baranska M. (2015). Raman spectroscopy of lipids: A review. J. Raman Spectrosc..

[18] Notingher I., Hench L.L. (2006). Raman microspectroscopy: A noninvasive tool for studies of individual living cells in vitro. Expert Rev. Med. Devices.

[19] Tsikritsis D., Richmond S., Stewart P., Elfick A., Downes A. (2015). Label-free identification and characterization of living human primary and secondary tumour cells. Analyst.

[20] Lee L.C., Liong C.Y., Jemain A.A. (2018). Partial least squares-discriminant analysis (PLS-DA) for classification of high-dimensional (HD) data: A review of contemporary practice strategies and knowledge gaps. Analyst.

[21] Candeloro P., Grande E., Raimondo R., Di Mascolo D., Gentile F., Coluccio M.L., Perozziello G., Malara N., Francardi M., Di Fabrizio E. (2013). Raman database of amino acids solutions: A critical study of extended multiplicative signal correction. Analyst.

[22] De Gelder J., De Gussem K., Vandenabeele P., Moens L. (2007). Reference database of Raman spectra of biological molecules. J. Raman Spectrosc..

[23] Pyrak E., Jaworska A., Kudelski A. (2019). SERS Studies of Adsorption on Gold Surfaces of Mononucleotides with Attached Hexanethiol Moiety: Comparison with Selected Single-Stranded Thiolated DNA Fragments. Molecules.

[24] Kelly J.G., Najand G.M., Martin F.L. (2011). Characterisation of DNA methylation status using spectroscopy (mid-IR versus Raman) with multivariate analysis. J. Biophotonics.

