# Supplementary material for: Subtyping on Live Lymphoma Cell Lines by Raman Spectroscopy
Source: Materials (Basel). 2022 Jan 12;15(2):546. doi: 10.3390/ma15020546 (PMC8778083; doi:10.3390/ma15020546)
Supplement: Supplementary file 1 [file materials-15-00546-s001.zip › materials-1528303-supplementary.pdf]

materials

MDPI

Supplementary Materials

Subtyping on Live Lymphoma Cell Lines by Raman Spectroscopy

Klytaimnistra Katsara [1,2], Konstantina Psatha [1], George Kenanakis [3], Michalis Aivaliotis [1,†,‡] and Vassilis M. Papadakis [1,*]

[1] Institute of Molecular Biology and Biotechnology, Foundation for Research and Technology-Hellas, N. Plastira 100, GR-70013 Heraklion, Greece; klytaimnistra_katsara@imbb.forth.gr (K.K.); konstantina_psatha@imbb.forth.gr (K.P.); aivaliot@imbb.forth.gr (M.A.)
[2] Department of Chemistry/Biochemistry Section, University of Crete, Andrea Kalokerinou, GR-71500 Heraklion, Greece
[3] Institute of Electronic Structure and Laser, Foundation for Research and Technology-Hellas, N. Plastira 100, GR-70013 Heraklion, Greece; gkenanak@iesl.forth.gr
* Correspondence: vassilis_papadakis@imbb.forth.gr; Tel.: +30-28-1039-1267
† Current address: Laboratory of Biochemistry, Department of Medicine, School of Health Sciences, Aristotle University of Thessaloniki, GR-54124 Thessaloniki, Greece.
‡ Current address: Functional Proteomics and Systems Biology (FunPATh), Center for Interdisciplinary Research and Innovation (CIRI-AUTH), Balkan Center, GR-54124 Thessaloniki, Greece.

Citation: Katsara, K.; Psatha, K.; Kenanakis, G.; Aivaliotis, M. Subtyping on Live Lymphoma Cell Lines by Raman Spectroscopy. *Materials* **2022**, *15*, 546. https://doi.org/10.3390/ma15020546

Academic Editor: Francesco Inchingolo

Received: 12 December 2021
Accepted: 9 January 2022
Published: 12 January 2022

Publisher's Note: MDPI stays neutral with regard to jurisdictional claims in published maps and institutional affiliations.

Copyright: © 2022 by the authors. Licensee MDPI, Basel, Switzerland. This article is an open access article distributed under the terms and conditions of the Creative Commons Attribution (CC BY) license (https://creativecommons.org/licenses/by/4.0/).

2. Materials and methods

2.2.5 - Raman spectral database

Through the analysis of the Raman signals and the peaks identified during the experiments, a database was constructed. Raman signal assignments derived from literature, in order to translate Raman spectral findings to biological information. In the left column of the table, the Raman peak wavenumber is presented. When a temperature dependence was observed in the Raman peaks, an asterisk was placed next to the corresponding wavenumber for discrimination. In the second column the Raman peak assignments are presented. Additionally, to the asterisk, a color coding (red color) is used to indicate the temperature-based Raman peaks and assignments.

Table S1. Total list of Raman peak assignments. In red color and with an asterisk in superscript the temperature dependent Raman peaks are presented.

Wavenumber (cm^{-1})	Assignment
601,602	Nucleotide conformation
745*	700–745 cm^{-1} ➔ ν(C-S) trans (amino acid methionine)
747,748,749*	747 cm^{-1} ➔ CH_2 rocking, L-Phenylalanine [1] , 748 cm^{-1} ➔ DNA, ring breathing of pyrimidine of T [2], 749 cm^{-1} ➔ Symmetric breathing of tryptophan
779, 782,783	DNA/RNA, 780 cm^{-1} ➔ Uracil-based ring breathing mode, 782,783 cm^{-1} ➔ Phosphodiester, Cytosine, Thymine, Uracil
999	ν_{45}(C-C), observed in the spectra of single human Red blood cell (RBC)
1000,1001,1002	1000 cm^{-1} ➔ Phenylalanine, Bound and free NADH, 1001 cm^{-1} ➔ Symmetric ring breathing mode of phenylalanine, 1002 cm^{-1} ➔ C-C aromatic ring stretching, Phenylalanine
1008	Proteins (L-Serine) [1], Phenylalanine, ν(C-O), ν(C-C)
1091	1090 cm^{-1} ➔ Symmetric phosphate stretching vibrations, 1092–1093 cm^{-1} ➔ Phosphodioxy
1125-1134*	Adenine (1125, 1134 cm^{-1}[1]) and ν(C-N) stretching (lipids, 1127 cm^{-1} ➔ proteins), ν(C-C) skeletal of acyl backbone in lipid (1129 cm^{-1} ➔ trans conformation), Phospholipid structural changes (trans versus gauche isomerism), Acyl chains, Palmitic acid
1191, 1194	1191 cm^{-1} ➔ L-Valine, acetoacetate, 1194 cm^{-1} ➔ L-Proline [1], 1185–1300 cm^{-1} ➔ Antisymmetric phosphate vibrations
1251	Guanine, cytosine (NH_2)
1298	1220-1300 cm^{-1} ➔ Amide III (arising from coupling of C-N stretching & N-H bonding-can be mixed with vibrations of side chains), amide III (L-Arginine [1]), CH bend in Lipids (Palmitic acid), Acyl chains
1310	1309 cm^{-1} ➔ CH_3/CH_2 twisting or bending mode of lipid/collagen
1312	1313 cm^{-1} ➔ CH_3CH_2 twisting mode of collagen/lipid
1333	DNA/RNA, Guanine, Acetyl coenzyme A [1]
1334-1341*	Polynucleotide chain (1334 cm^{-1} ➔ DNA/RNA purine bases, Guanine, Adenine), proteins (1337 cm^{-1} ➔ amide III, CH_2 wagging vibrations from Glycine backbone and proline side chain, L-Histidine, L-Tryptophane, L-Glutamate), CH_3CH_2 wagging mode of collagen, CH protein deformation
1373,1375	T, A, G (ring breathing modes of the DNA/RNA bases), Acetyl coenzyme A [1]
1435	Thymine (weak) [1], 1420-1481 cm^{-1} ➔ DNA/RNA, Guanine, Adenine, 1436 cm^{-1} ➔ CH_2 scissoring (lipid band)
1441	CH_2 scissoring and CH_3 bending in lipids, Cholesterol and its esters, C-H bending mode of accumulated lipids in the vecrotic core of the atheromatous plaque
1445,1446,1447	δ(CH_2), δ(CH_3) protein (collagen) and lipid (phospholipids) assignment (CH_2 bending mode being of diagnostic significance), CH_2 deformation (1446 cm^{-1}), 1447 cm^{-1} ➔ CH_2 deformation (protein vibration)-A marker for protein concentration

1484	1480–1575 cm^{-1} ➔ Amide II (largely due to a coupling of C-N stretching and in-plane bending of the N-H group<br>1485 cm^{-1} ➔ G, A (ring breathing modes in the DNA bases) Nucleotide acid purine bases (guanine and adenine), Purine rings (guanine) [3]<br>1483 cm^{-1} ➔ CG (C4 me) associated with CH_2 and CH_3 bending and stretching vibrations, CG (C4 me) [3]
1502	1499 cm-1 ➔ C-C stretching in benzenoid ring
1509	1510 cm^{-1} ➔ Cytosine, A (ring breathing modes in the DNA bases
1578,1581-1586*	1586 cm^{-1} ➔ DNA/RNA (1578 cm^{-1} ➔ Guanine (N3), Guanine, adenine), Phenylalanine, hydroxyproline (1582 cm^{-1} ➔ C=C stretching)
1604	1600–1800 cm^{-1} ➔ Amide I band of proteins; due to C=O stretching Amide I (which is due mostly to the C=O stretching vibrations of the peptide backbone; has been used the most for structural studies due to its high sensitivity to small changes in molecular geometry and hydrogen bonding of peptide group)<br>1602 cm^{-1} ➔ Phenylalanine, δ(C=C), phenylalanine (protein assignment)<br>1603 cm^{-1} ➔ C=C in-plane bending mode of phenylalanine and tyrosine, Ring C-C stretch of phenyl<br>1605 cm^{-1} ➔ Cytosine (NH_2)
1650-1656	1650 cm^{-1} ➔ amide I (C=C) absorption, 1653 cm^{-1} ➔ Lipid (C=C stretch, Ascorbic acid [1]) 1656 cm^{-1} ➔ cis phospholipids, Carbonyl stretch (C=O)
1672	C=C stretch, Amide I band (C=O stretch coupled to a N-H bending)
2851,2852	CH_3 symmetric stretch of lipids
2869	CH_2 asym stretches and CH stretches in lipids and proteins, CH_2 symmetric stretch of lipids
2919	CH_2 asym stretches and CH stretches in lipids and proteins, CH_2 asymmetric stretch of lipids and proteins, CH_3 stretching vibration
2928	CH_2 asym stretches and CH stretches in lipids and proteins, Symmetric CH_3 stretch due primarily to protein
2934,2938	2934 cm^{-1} ➔ CH_2 asym stretches and CH stretches in lipids and proteins (2938 cm^{-1})
2947,2949, 2950	CH_3 stretching vibrations

2.2.6 – Raman spectra processing and analysis

Background was subtracted from the raw Raman spectra as shown in the following typical example in (Figure S1).

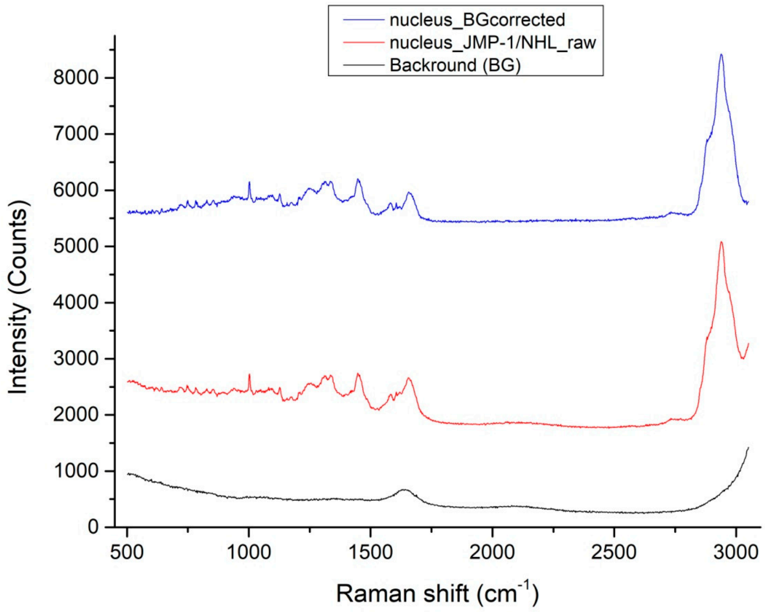

Figure S1. Demonstration of the Raman spectral background subtraction. Spectra are presented with an offset between them, to allow better visualization of the spectral background changes.

3. Results and Discussion

3.1. JMP-1/MCL NHL and MDA-V/HL cell lines comparison

We repeated the PCA by performing two main processing tasks. One using mean centering and second performing variance adjustment. Results showed no significant improvement. In the following Figure S2 and Figure S3 we present the PCA results.

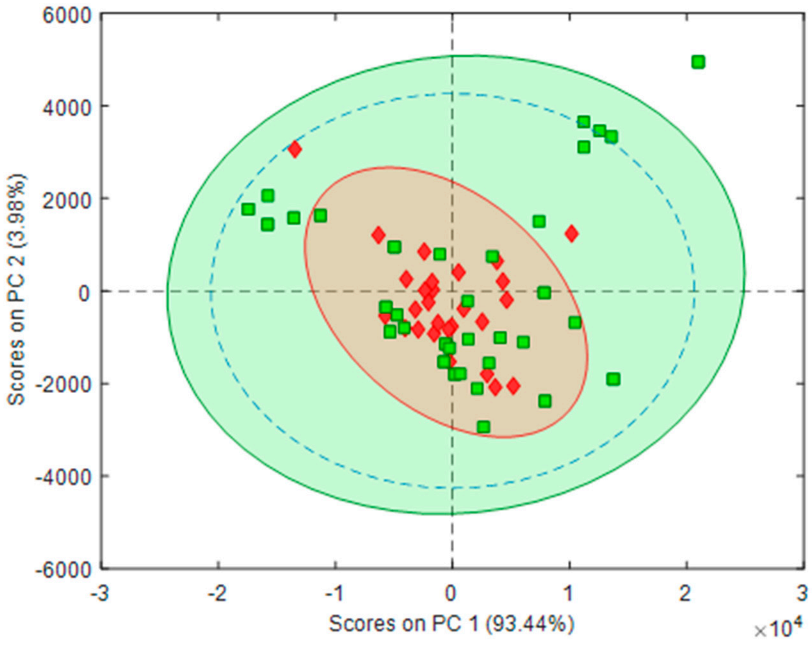

Figure S2. PCA of PC1 and PC2 JMP-1/MCL NHL and MDA-V/HL cell lines comparison.

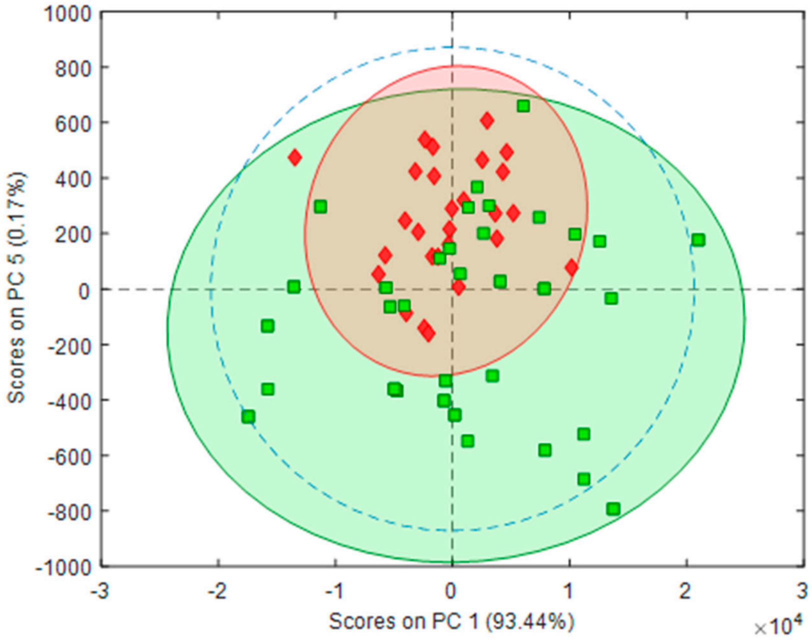

Figure S3. PCA of PC1 and PC2 JMP-1/MCL NHL and MDA-V/HL cell lines comparison.

3.2. Comparison of experiments BR1 vs. BR2 of JMP-1/MCL cell line

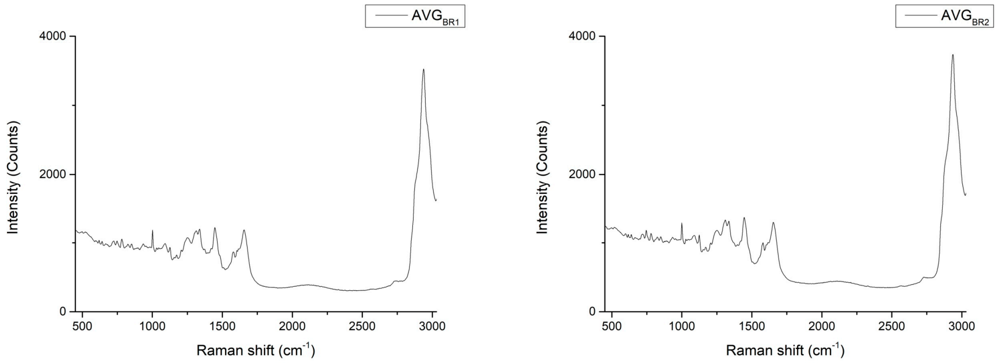

Figure S4. S: Average Raman spectra from BR1 (left) and BR2 (right) (JMP-1/NHL) experiments.

S3.5. Cell line differences due to temperature

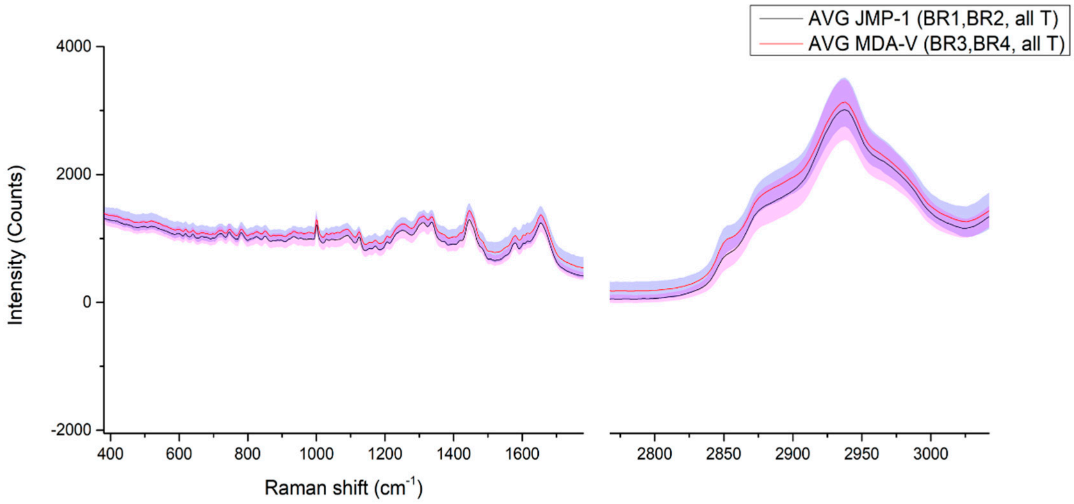

Figure S5. S: Average Raman spectra from J-MP1 and MDA-V cell lines from all measurements in all Temperatures. In shadow the related SD of each average is presented.

Table S2. S: Major Raman bands identified as significant under different temperatures for the MDAV/HL cell line. In red color and with a star superscript the temperature dependent Raman peaks are presented.

MDA-V/HL Differences					
	Wavenumber shift range (cm-1)				
**Temp difference**	**749**	**1008**	1125 - 1134	1581 - 1586	1604
T30 - T37	21	22	15	24	49
T25 - T37	26	20	26	36	48
T20 - T37	26	34	27	31	43
T18 - T37	16	42	22	31	47
T15 - T37	14	41	26	40	52

Table S3. S: Major Raman bands identified as significant under different temperatures for the JMP1/MCL cell line. In red color and with a star superscript the temperature dependent Raman peaks are presented.

JMP-1/MCL Differences					
	Wavenumber shift range (cm-1)				
**Temp difference**	**745 – 748**	**999 - 1001**	1333 - 1341	1441 - 1446	2934
T30 - T37	12	30	24	74	426
T25 - T37	23	39	17	74	429
T20 - T37	25	31	27	42	430
T18 - T37	20	44	20	74	406
T15 - T37	19	49	16	66	428

3.6 – Bright field cell imaging

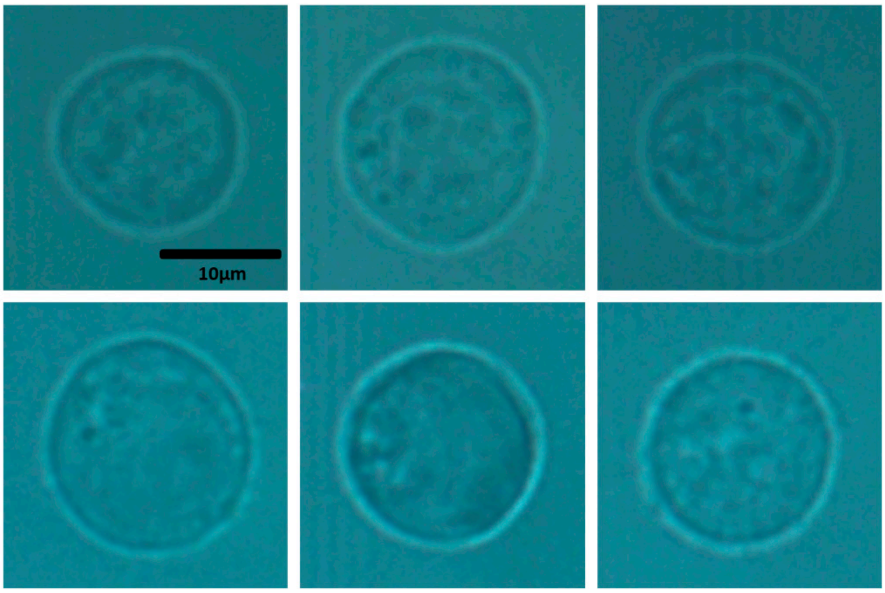

Figure S6. S: Randomly selected cell images from bright-field microscope. In the upper and bottom line JMP-1/NHL and MDA-V/HL cell line images are presented respectively, monitoring indistinguishable morphological differences following a successful completion of the measurement.

Author Contributions: Conceptualization and methodology, V.M.P.; bibliographic investigation, K.K., K.P., M.A., G.K., and V.M.P.; experimental characterization and analysis, K.K., K.P., G.K., and V.M.P.; writing—review and editing, K.K. and V.M.P.; supervision, M.A. and V.M.P.; funding acquisition K.P. and V.M.P. All authors have fully read and approved the final version of the manuscript.

Funding: This work was financially supported by the project BIOIMAGING-GR (MIS 5002755) implemented under "Action for Strengthening Research and Innovation Infrastructures," funded by the Operational Programme "Competitiveness, Entrepreneurship and In-novation" (NSRF 2014–2020) and co-financed by Greece and the European Union (European Regional Development Fund) (V.M.P.), and the Stavros Niarchos Foundation within the framework of the project ARCHERS ("Advancing Young Researchers' Human Capital in Cutting Edge Technologies in the field of Systems Biology Approaches and Personal Genomics for Health and Disease Treatment") (K.P. and V.M.P.).

Institutional Review Board Statement: Not applicable.

Informed Consent Statement: Not applicable.

Data Availability Statement: All data are available upon request. The data presented in this study are available on request from the corresponding author.

Acknowledgments: The authors would also like to thank Dimitrios Tsikritsis for the fruitful discussions and his validation of the results and Elias Drakos for providing the cell lines.

Conflicts of Interest: The authors declare no conflict of interest.

References

1. De Gelder, J., et al., *Reference database of Raman spectra of biological molecules.* Journal of Raman Spectroscopy, **2007**. **38**: p. 1133-1147.
2. Pyrak, E., A. Jaworska, and A. Kudelski, SERS Studies of Adsorption on Gold Surfaces of Mononucleotides with Attached Hexanethiol Moiety: Comparison with Selected Single-Stranded Thiolated DNA Fragments. Molecules, **2019**. **24**, 3921. https://doi.org/10.3390/molecules24213921.
3. Kelly, J.G., G.M. Najand, and F.L. Martin, *Characterisation of DNA methylation status using spectroscopy (mid-IR versus Raman) with multivariate analysis.* J Biophotonics, **2011**, *4*, 345-54.
